# Integrated Land Suitability Assessment for Depots Siting in a Sustainable Biomass Supply Chain

**DOI:** 10.3390/s23052421

**Published:** 2023-02-22

**Authors:** Ange-Lionel Toba, Rajiv Paudel, Yingqian Lin, Rohit V. Mendadhala, Damon S. Hartley

**Affiliations:** 1 System Dynamics & Modeling, Idaho National Laboratory, Idaho Falls, ID 83415, USA; 2 Operations Research and Analysis, Idaho National Laboratory, Idaho Falls, ID 83415, USA; 3 Geospatial Data Science & Applications, Idaho National Laboratory, Idaho Falls, ID 83415, USA

**Keywords:** supply chain analysis, geospatial modeling, suitability analysis, ecological factors, transportation network analysis, woody crop

## Abstract

A sustainable biomass supply chain would require not only an effective and fluid transportation system with a reduced carbon footprint and costs, but also good soil characteristics ensuring durable biomass feedstock presence. Unlike existing approaches that fail to account for ecological factors, this work integrates ecological as well as economic factors for developing sustainable supply chain development. For feedstock to be sustainably supplied, it necessitates adequate environmental conditions, which need to be captured in supply chain analysis. Using geospatial data and heuristics, we present an integrated framework that models biomass production suitability, capturing the economic aspect via transportation network analysis and the environmental aspect via ecological indicators. Production suitability is estimated using scores, considering both ecological factors and road transportation networks. These factors include land cover/crop rotation, slope, soil properties (productivity, soil texture, and erodibility factor) and water availability. This scoring determines the spatial distribution of depots with priority to fields scoring the highest. Two methods for depot selection are presented using graph theory and a clustering algorithm to benefit from contextualized insights from both and potentially gain a more comprehensive understanding of biomass supply chain designs. Graph theory, via the clustering coefficient, helps determine dense areas in the network and indicate the most appropriate location for a depot. Clustering algorithm, via K-means, helps form clusters and determine the depot location at the center of these clusters. An application of this innovative concept is performed on a case study in the US South Atlantic, in the Piedmont region, determining distance traveled and depot locations, with implications on supply chain design. The findings from this study show that a more decentralized depot-based supply chain design with 3depots, obtained using the graph theory method, can be more economical and environmentally friendly compared to a design obtained from the clustering algorithm method with 2 depots. In the former, the distance from fields to depots totals 801,031,476 miles, while in the latter, it adds up to 1,037,606,072 miles, which represents about 30% more distance covered for feedstock transportation.

## 1. Introduction

The agricultural supply chain refers to the supply chain for any type of agricultural product such as dairy, grain, vegetable, fruit, or biomass feedstock. It normally starts from agricultural production and ends at distribution to customers, and it may include many actors such as farmers, suppliers, researchers, distributors, customers, and stakeholders. The sustainability of agricultural supply chains is about achieving maximal performances in economic growth, environmental protection, and social development [1]. To ensure the sustainability of agricultural supply chains, it is critical to improve farmers’ willingness to participate in marketing and coordination strategies and support the reliability of product supply [2]. Other key factors influencing sustainability include farm inputs, energy use efficiency, waste management, and logistical efficiencies during crop production, harvesting, storage, and handling [3].

Different frameworks and metrics have been developed and proposed to measure the sustainability (social, environmental, and economic) of agricultural supply chains. For example, social dimensions of an agricultural supply chain include aspects such as job creation [4], benefits to rural communities [5,6], health and safety practices [7], and community initiatives [8]. Environmental concerns are generally related to heavy chemicals (fertilizers and herbicides) and fossil fuel usage in farming practices, which lead to environmental degradation and public health issues [9]. Soil quality and soil carbon dynamics have also been identified as important metrics in measuring the sustainability of a crop production system [10], as well as soil carbon sequestration. Techniques such as reduced tillage [11], on-farm compost [12], cover crops [13,14] have been evidenced to affect soil health. Land suitability analysis has also helped inform decision making on environmental health [15,16]. Metrics that evaluate economic sustainability are generally revenue-oriented [1]. Key indicators in measuring economic sustainability or viability include profitability, liquidity, stability, and productivity [17]. Profitability measures the difference or ratio between the revenue generated and the cost of inputs used, liquidity indicates the return-on-investment term, stability is estimated by the share and development of capital investment, and productivity measures the ratio of input and out or production efficiency of a certain system. In a biofuel supply chain, the economic performance indicators are generally defined as cradle-to-reactor production costs [18] and potential savings from greenhouse gas emission reductions [19].

Integrated landscape management (ILM) has emerged as a strategy to integrate biomass production practices into agricultural production fields to increase agricultural supply chain sustainability [20]. Applicable field areas include low-yielding zones where soil properties are not conducive to row crop production or areas where high crop productivity generates excess crop residues that benefit from biomass residue harvest and collection. ILM can be viewed as an improvement over current agricultural management schemas that are wholly dependent upon monoculture agriculture and do not account for subfield variability. In this paper, we are interested in the application of ILM for biomass feedstock production supply chain sustainability via the simultaneous production of agricultural and woody crops on the same land parcel.

Integrating short-rotation woody crops (SRWC) such as poplars and shrub willows into the agricultural landscape has been demonstrated to result in many environmental and economic benefits such as water quality improvement (nutrient loading reduction), soil carbon sequestration [21], wildlife habitat benefits [22], nitrate leaching reduction [23,24] as well as GHG emissions reduction [25,26]. In addition, these woody crops are economically viable on lands marginal for row-crop production and able to produce more yield than normal hardwood species [27].

Several research papers have investigated how biomass supply chains can be organized in a cost-efficient manner to collect, process, and transfer available biomass to a specific biorefinery plant [28,29,30,31]. One of the main methods used is mathematical programming (or optimization theory), including linear programming (LP), nonlinear programming (NLP), mixed integer linear programming (MILP), and mixed integer nonlinear programming (MINLP) models [32,33,34,35,36,37,38,39,40,41]. Heuristics and meta-heuristic techniques have also been employed [42,43,44,45]. Although not necessarily providing optimal solutions, heuristics explores near-optimal solutions to complex problems in a quicker manner and with less computational demand. These approaches are combined with the geographic information system (GIS) to capture spatial and temporal information to conduct biofuel logistics studies. Simulation modeling is also used [46,47,48,49,50,51] to assess biomass supply chain operations under various scenarios, including the uncertainties and variabilities that exist in systems.

Most of these studies only consider the economic aspect, that is, minimizing the total costs related to facilities, transportation, harvesting, and collection. There has also been a growing interest in incorporating environmental objectives to biomass supply chain analysis. However, environmental impacts have only been quantified in terms of CO_2_ emission due to transportation and biorefinery operations [52,53,54,55]. Although costs and GHG emission are important in developing a sustainable biomass supply chain, site suitability through ecological indicators is as important and needs to be accounted for. Soil characteristics or ecological indicators are less often investigated, yet critical in planning for a sustainable biomass supply, as it dictates feedstock production. The chain starts from raw materials supply and terminates with end customers thanks to various processing and movement across locations. Without feedstock supply, there can be no bioenergy, and this supply is only made possible within the constraints of acceptable soil features.

In this study, we aim to analyze biomass supply chain sustainability by considering both economic and environmental aspects, using *suitability* rather than *optimality* only, as opposed to the current literature. Economic impacts are captured via the distances traveled. Shorter distances would incur lower costs and generate lower emissions, with potential savings. Environmental impacts are captured via ecological indicators, gauging suitability for SRWC. Practically, our research investigates field-level design for polyculture landscapes with both agricultural crop and woody biomass feedstock crop production with environmental and economic sustainability. This method consists of integrating (1) a field suitability model for energy crop production, providing agricultural fields scoring based on soil characteristics relevant to SRWC, and (2) a transportation network model, also providing scoring based on pre-processing or depot location distance to fields.

The next section details the data used and the method developed, including problem characterization and methodology. Section 3 presents the results obtained, while Section 4 provides conclusions.

## 2. Materials and Methods

### 2.1. Problem Characterization

**Figure 1:**
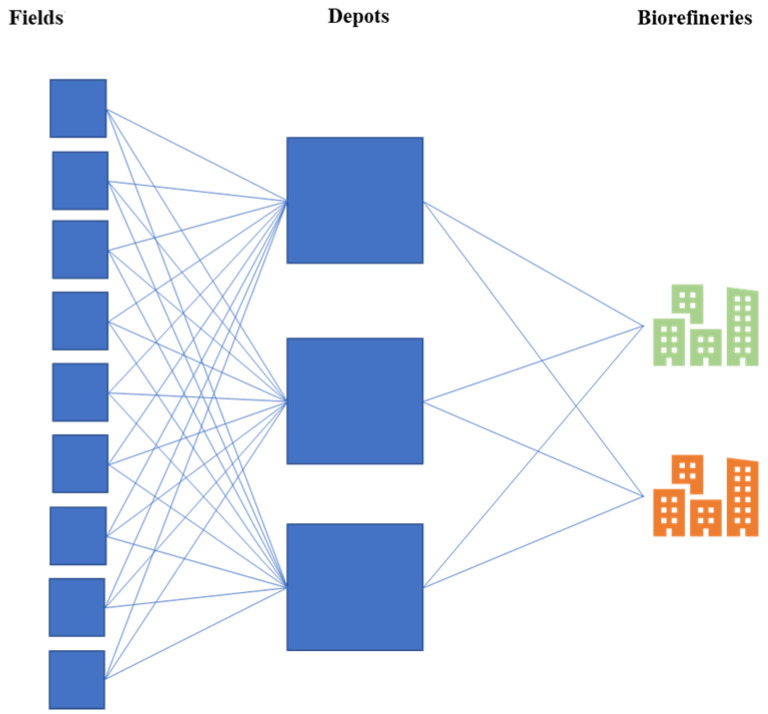
Schematic representation of the supply system.

We define the feedstock supply system as a system infrastructure for the collection, transportation, and transformation of feedstock for bioenergy production. The objective is to manage the flow of material and information in the chain of supply in a way that will provide the highest cost-effective and environmentally friendly advantage. The idea is to move feedstock from the fields to the closest preprocessing depots, and later to biorefineries for conversion. Thin blue lines represent the transportation network system (Figure 1). Note that the lines indicate potential transport connections, as fields are not necessarily all connected to multiple depots.

**Figure 2:**
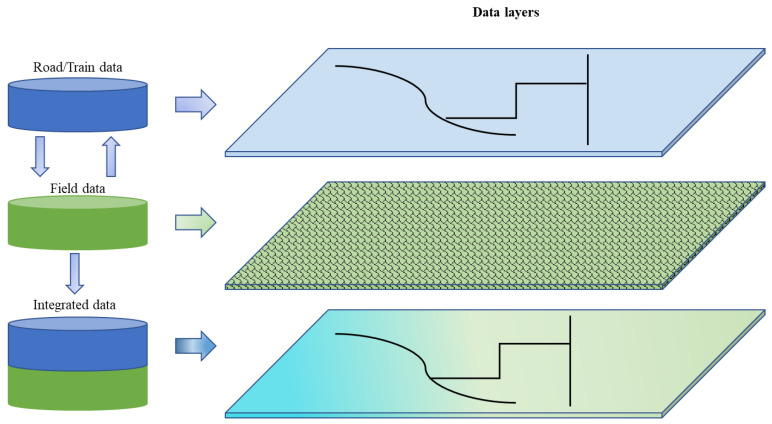
Integrated data layers.

We consider the integration of 2 layers: fields and transportation network layers (Figure 2). The fields layer encompasses data related to agricultural fields, including shape, geographical details (latitude and longitude), field suitability index, ecological indexes, and acreage. The transportation network layer encompasses data related to road and rail networks, including intersections and distances, enabling transportation of feedstock from fields to depots, and biorefinery. Note, however, that the focus of this study is more on depot location. Both layers exchange data, with the conversion of field data into road data, and vice versa. This conversion is explained in Section 2.2.2.

The integrated layer thus constitutes the location of the depots, as well as a biorefinery. The number of depots and their locations is determined based on proximity to the fields so as to minimize feedstock distance traveled and field suitability to SRWC. The location of the biorefinery is assumed at the most populous area in the region considered for analysis. The rationale behind this selection is that such a populated area can serve as a workforce.

### 2.2. Methodology

**Figure 3:**
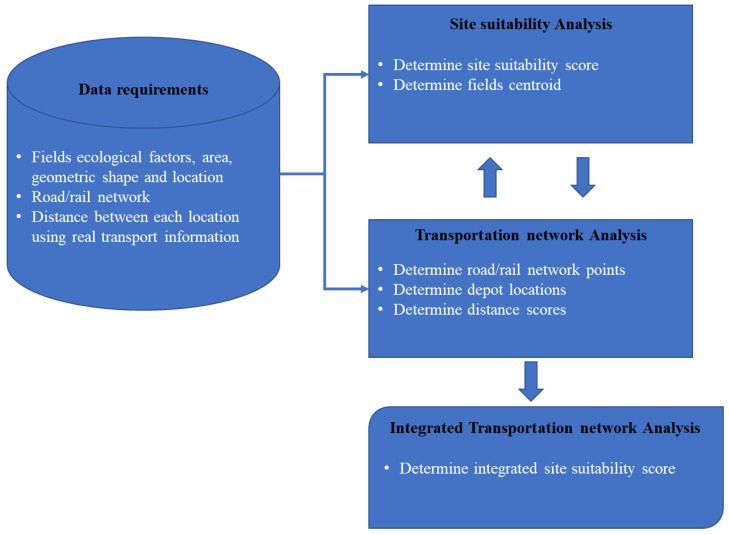
Methodology schematic.

The methodology (Figure 3) consists of developing an integrated site suitability analysis, incorporating the SRWC feedstock along with the transportation network to score agricultural fields, for the potential to sustainably supply biomass. Data used include geospatial field information, as well as road/rail networks.

The first step is the site suitability analysis, consisting of scoring fields based on ecological/environmental factors. These factors are defined in Section 2.2.1. The scores help identify the best locations for an activity, in our case, SRWC production. Site suitability modeling is a widely used approach for these questions [56,57,58], factoring in multiple factors, different in importance, highlighting locations that best meet selected criteria for said site. The scores are normalized to a 0–1 scale. The fields’ centroids are also determined, to be used in the transportation network analysis. The second step is the transportation analysis. As feedstock is produced on fields, it needs to be processed, first through depots, and then through biorefineries, using road and rail networks as their transportation means. The selection of depots is performed with the goal of reducing costs and negative environmental implications. We look to find the closest routes/distances from each field to the depot locations. All distances are then normalized to a 0–1 scale, 1 scoring the fields that are closest from the depots and 0 the farthest.

The final step is the combination of both scoring values. This integrated site suitability index (SSI) is computed by taking the average of the initial SSI and normalized depot distance values.

#### 2.2.1. Site SUITABILITY ANALYSIS

Site suitability modeling is used to identify, qualify, compare, and rank candidate pixels that are more appropriate for a certain crop. Each factor is standardized to a value between 0 and 1, 1 being the most suitable, and 0 being the least suitable. These factors selected are specific to SRWC and woody biomass site suitability analysis. See Table 1 for details about these factors and their data sources.

The site suitability value for each field is calculated using a linear fuzzy logic prediction model developed by Wu, et al. [59] and shown in the equation below:

(1) $$SSI_{i}=\overset{n}{\underset{k=0}{\sum }}f_{mi}w_{m}\times \prod b_{n}$$

where SSI for field *i* is the site suitability index, *f_m_* is the fuzzy value of criteria *m* for field *i*, *w_m_* is the weight of criteria m, *b_n_* is the criteria score of constraint n (binary value), and ∏ is the product. Binary values (0 and 1) were assigned to land cover and slope. Fuzzy logic membership functions were built to determine the fuzzy value of the other criteria, including water availability, soil productivity, soil texture, and erodibility factor. The final calculation normalizes the site suitability values to a range of 0 and 1 based on the weighting values. For this analysis, all weighting values were set to 1.

**Table 1:** Ecological/environmental factors considered for SRWC.

Factors	Data/Assumptions
Land cover/crop rotation: SRWC helps improve soil attributes, reduce soil erosion, and sequester soil organic carbon [60]. ILM SRWC land suitability analysis mainly targets low-productivity agricultural fields. This ensures energy production and environmental protection without compromising food production.	USDA National Agriculture Statistics Services (NASS) data obtained from https://nassgeodata.gmu.edu/CropScape/ (accessed on 26 August 2022). The crop data layer from 2018, which consists of 30 m resolution raster data, was converted to vector fields. Field-level cultivation information was also obtained from the CDL layer and a subset of cultivable area larger than 100 acres was considered for the analysis.
Slope: Growing SRWC on slopes helps in stabilizing land, reducing runoff, and controlling soil erosion. However, steep slopes could be problematic for equipment operations. A slope > 8% is less desirable for SRWC due to difficulties in using harvesting equipment [61,62].	National elevation data (NED) for slope information were obtained from https://www.usgs.gov/the-national-map-data-delivery/gis-data-download (accessed on 26 August 2022). From the NED data, we only considered fields with slopes less than 8%.
Soil Productivity: We used the National Commodity Crop Productivity Index (NCCPI). This index was developed by the USDA to estimate commodity crop (i.e., corn, soybeans, cotton, or small grains) productivity in non-irrigated agricultural land [63]. Since we prioritize low-productive agriculture fields for SRWC production, areas having low NCCPI values are prioritized.	Gridded Soil Survey Geographic (gSSURGO) Database was used and obtained from https://data.nal.usda.gov/dataset/gridded-soil-survey-geographic-database-gssurgo (accessed on 26 August 2022).
Soil texture: Soil texture is important for the retention of soil moisture as well as plant root growth. Pinno and Belanger [64] study found soil texture to be the best predictor of tree growth.	Data used were from SSURGO datasets. (https://www.nrcs.usda.gov/wps/portal/nrcs/main/soils/survey/, accessed on 26 August 2022). We prioritized land with high silt and clay for woody biomass production.
Soil erodibility factor: Soil erosion is the result of inadequate soil management and constitutes a major threat to the productivity and sustainability of crops [65]. We used the K factor.	Data used were from SSURGO datasets. (https://www.nrcs.usda.gov/wps/portal/nrcs/main/soils/survey/, accessed on 26 August 2022).
Water Availability: Water is essential for plant growth. In dry and non-irrigated environments, soil moisture is extremely important for the growth of SRWC [66,67]. We used the soil availability water storage (AWS) index.	

#### 2.2.2. Transportation Analysis

This analysis is conducted to determine the locations of depots using transportation networks. The road network is used for transportation from fields to depots. For implementation, we present 2 methods: clustering analysis and graph theory. Depots are assumed to be located at the center of spatial or graph-based clusters. We use OSMnx, a Python package to model, project, visualize, and analyze real-world street networks as well as geospatial geometries [68]. Other Python libraries used are Networkx, for the creation and analysis of the structure and dynamics of networks, represented in the form of graphs with nodes and edges [69], and GeoPandas, Shapely, Rasterio, and Rasterstats to manipulate geospatial data and allow spatial operations [70,71,72].

The methods employed essentially help translate information from the fields layer into information for the transportation network layer, and vice versa. In both methods, each field centroid is tied to, or paired with, the closest node on the road network in terms of distance. For our study, we used Euclidean distance, which is the straight-line distance *d* in parameter space between two points of coordinates (*x*_1_, *y*_1_) and (*x*_2_, *y*_2_), given by the following equation [73]:

(2) $$d=\sqrt{{\left(y_{2}-y_{1}\right)}^{2}+(x_{2}-x_{1}{)}^{2}}$$

Just like fields are scored for their suitability, distances from fields to depots, using road networks, are also scored. These distances are normalized to obtain a 0 to 1 score, with 1 for the closest and 0 for the farthest.

##### Graph Theory

Graph theory helps describe the characterization of the transportation network. Graphs formally represent a network, which is basically composed of vertices (or nodes) that are connected by edges (or links). Graph theory provides meaningful information about the topological architecture of the networks at hand [74]. Applied to transportation, it helps quantify levels of modular organization and the connectedness of field locations for depot selection. The most connected/accessible location ranks the highest to be a depot. As we intend to identify connectivity patterns in the transportation network to identify the most suitable depot siting, this approach is appropriate. More information about graph theory can be found in [75,76].

For this analysis, we used the clustering coefficient, which represents “the degree to which nodes in a graph tend to cluster together” [77]. By nodes, we mean road intersections. This metric represents the level of connectivity in or density of the network. A dense network is a network in which each node is linked to almost all other nodes, while in a sparse network, the number of connections is low. All values belong to 0–1 interval, 1 indicating the densest, and 0 indicating the least dense.

**Figure 4:**
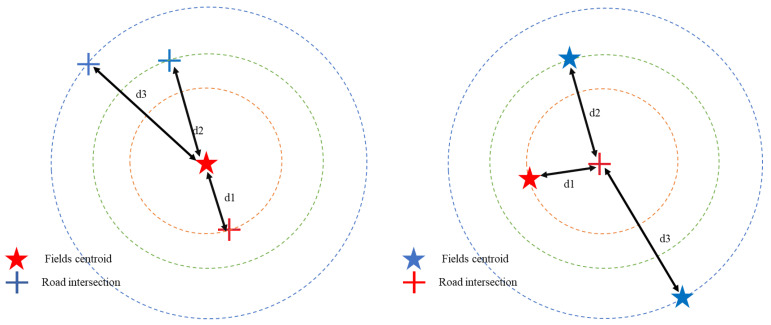
Data conversion of road nodes to centroids (**left**) and centroids to road nodes (**right**). Distances d1, d2, and d3 represent distances from centroids to road nodes, with d1 being the shortest one.

All nodes’ data information is converted into field data. The goal is to find the closest field centroids to the road nodes/intersections. Using the coordinates of the nodes and the centroids, we compute the Euclidean distance to find these centroids (Figure 4, right. The red star is the field identified as a depot candidate). This is to make sure all fields are accessible via the road network. The field’s centroid that is the closest to the node/intersection with the most connected nodes is identified as a depot candidate. Considering the large size of the area (defined in Section 2.3), several nodes had 1 as the clustering coefficient. We, therefore, used the preliminary SSI and service area radius for depot selection. Candidate depots with 1 as the clustering coefficient value are ranked based on the highest SSI value. The 1st selected is the field with the highest SSI score. The 2nd field, with the 2nd highest SSI, is selected if the distance between 1st and 2nd is greater than the service area radius. The cycle continues until there are no additional candidate depots outside the service area.

##### Clustering Analysis

Clustering is a popular machine learning technique that is a method of unsupervised learning used for statistical data analysis. We used K-means clustering (centroid-based clustering algorithm), which consists of sorting N items or observations into K groups/clusters, often to uncover a structure within a complex set of data [78]. Each of these points is assigned to a cluster based on its squared distance from the centroid of that cluster [79]. Applied to our transportation problem, it helps find the best depot locations to service a given set of fields. Depots are viewed as cluster centroids and field locations as the data to be clustered. As we intend to analyze connectivity patterns in the transportation network to identify the most suitable depot siting, this approach is also appropriate. More information about K-means can be found in [80].

For this analysis, our goal was to minimize the distance between the chosen depot location and field centroid. Mathematically, this comes down to maximizing the distance between, and minimizing the distance within a predetermined number of clusters, K, in Equation (3), as defined in Tan, et al. [81]:

(3) $$E=\underset{j}{\sum }\underset{i}{\sum }{\left(x_{ij}-x_{j}\right)}^{2}$$

where *E* is the squared error function, *x_ij_* is an item (here field) *i* assigned to cluster *j*, and *x_j_* is the centroid of cluster *j*. The objective is to minimize E. Evidently, clusters need to be distinct from each other [82]. In our case, clustering (clustering algorithm) is based on the core idea of the fields’ centroids being gathered into clusters based on distances. At different distances, different clusters will form. At the center of the cluster is a field centroid, representing the depot location. The number of depots is determined by the number of clusters formed. Unlike the previous method where road network data are converted to fields data, this approach presents the other way around. Field centroids data are converted to the road network, also using the Euclidian distance, to ensure all fields are accessible via road network (Figure 4, left. The red cross is the closest intersection to the depot candidate).

#### 2.2.3. Integrated Site Suitability Analysis

This step integrates the scores obtained from SSI (see Section 2.2.1) and distances (see Section 2.2.2) computed earlier. These scores are averaged out, with equal weighting values. The result is an updated/integrated SSI value taking into account these 2 factors. The idea is to prioritize fields that are closer to depots that also have high SSI values.

### 2.3. Study Area

**Figure 5:**
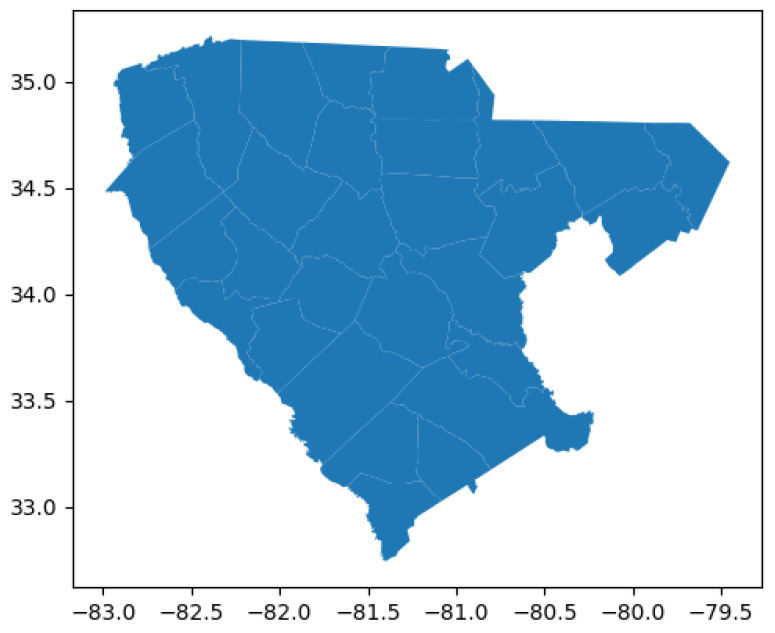
Geographical representation of the counties.

The area of interest is located in the Piedmont region, more specifically in South Carolina (Figure 5). South central/eastern US zones have been shown to be more prominent for SRWC [27,62,83]. This area is composed of 28 counties, including Abbeville, Greenwood, Laurens, Greenville, Chester, Spartanburg, Fairfield, Darlington, Newberry, Kershaw, Union, Lancaster, Chesterfield, Edgefield, McCormick, Barnwell, Aiken, Lexington, Saluda, Bamberg, Calhoun, Anderson, Orangeburg, Pickens, Richland, Cherokee, Allendale, Marlboro, and York. Fields in all these counties were captured using the USDA crop data layer from 2018.

## 3. Results and Discussion

**Figure 6:**
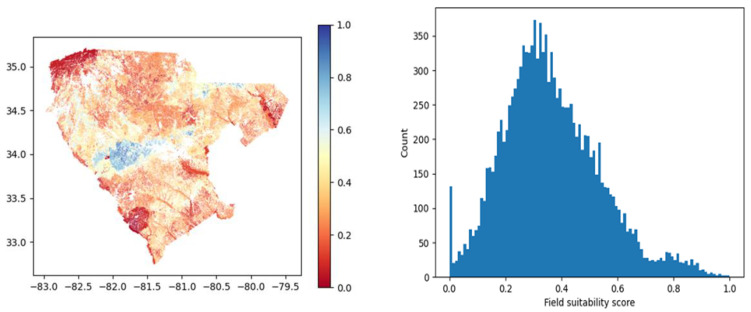
Map of fields (**left**) with suitability score for woody crops, where low-scoring fields are in the red spectrum and high-scoring fields in the blue spectrum. The histogram (**right**) shows the SSI distribution for all fields in the region.

A total of 13,063 fields spanning the 28-county region were scored using the criteria listed above. Figure 6 shows the SSI score distribution. The most suitable fields (highest SSI values) were located in the central area, with a good portion in the northeastern part. The northern and southern parts were the least suitable. The histogram shows that most fields have SSI values below 0.5, although most fields fall between 0.3 and 0.6. Looking at the map, fields in this scoring interval seem to be well spread, indicating an overall good suitability in the area.

**Figure 7:**
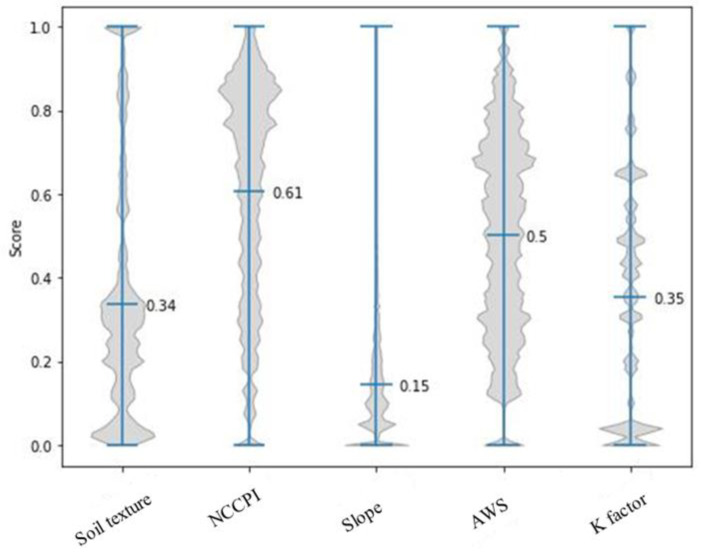
Violin plots showing data distributions of individual criteria with continuous scoring functions.

Figure 7 shows the distributions and median scores for individual criteria used to calculate the field SSI and presents a profile of the fields in our region of interest. NCCPI and AWS show the highest values, indicating the fields have, for the most part, high commodity productivity and good water storage capability. Good water availability would facilitate plant growth, which could ultimately enhance productivity. These characteristics show that the fields look profitable for woody crops.

As noted earlier, distances from fields (via closest road nodes) to depots were recorded using the road network. The notion of service area therefore becomes critical. In network analysis, service areas are areas surrounding selected objects, or base points, defining boundaries within which these selected objects can operate. In this study, a service area would thus indicate the delimited zone around a depot, accounting for all fields belonging to this zone, and model the movement of feedstock moving along the network in an organized and efficient manner. The objective is to reduce costs and limit the carbon footprint and is critical to ensure appropriate routing. A base point describes the location of the depot, whose accessibility is determined by a cut-off distance. We used a distance of 150 miles (240 km) as suggested by the Federal Motor Carrier Safety Administration (FMCSA) for a truck carrying agricultural products [84]. This represents a Euclidean distance specifying a radius within which the feasibility and profitability of feedstock supply are most likely. However, because we considered the road network (which was not built in a concentric fashion), the cut-off distance actually represented the maximum distance that can be traveled along the road network.

The integrated SSI (*intg_SSI*) value for each field was calculated using the equation we propose below:

(4) $$intg_{SSI}=\underset{m}{\sum }f_{m}w_{m}$$

where *intg_SSI_* for field *i* is the updated site suitability index, *f_m_* is the fuzzy value of a factor *m* for field *i*, and *w_m_* is the weight of factor *m*. Table 2 details the factors and the data sources.

**Figure 8:**
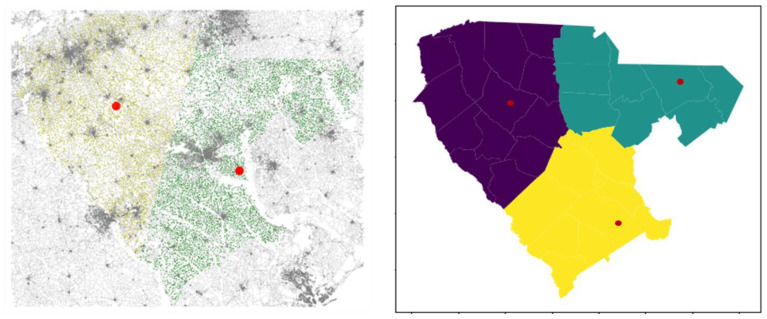
Candidate depot locations using K-means clustering with two field clusters (**left**) and using clustering coefficient with three field clusters (**right**). Red dots represent the locations of depots.

Figure 8 shows the depot locations for both *K-means clustering* and *graph theory* methods.

*K-means clustering*: The extent of the area studied was around 200 × 225 miles. After running the K-means algorithm, we determined two depots located in *Laurens* and *Richland* counties. Considering the service area radius, we submit that two depots should be sufficient and cost-effective to streamline the woody biomass supply system. Figure 8 (left) shows depot locations. The different colors specify the boundaries of the service area.

*Graph theory*: This approach analyzes the road network by determining network statistics and indicators. The clustering coefficient used here represents the extent to which a node’s neighborhood forms a complete graph [85]. It measures the degree to which nodes in a graph tend to cluster together, scaled to 0–1 interval. With several nodes indicating a value of 1, we chose the ones with the highest SSI scores of corresponding field centroids to those nodes. This was to prioritize areas with higher suitability. Given the area limits and service area radius, three depots were found, located in *Chesterfield*, *Laurens*, and *Orangeburg* counties. Figure 8 (right) shows depot locations, with here, too, different colors specifying the boundaries of the service area.

**Figure 9:**
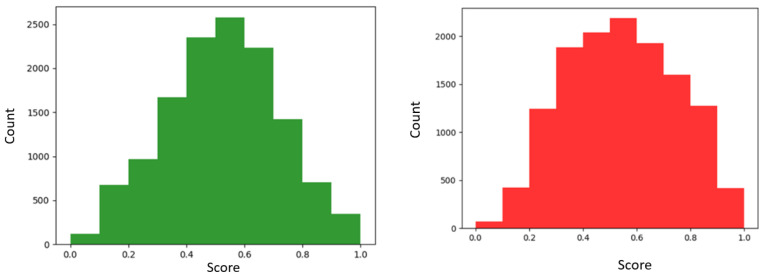
Transportation score using K-means (**left**) and graph theory (**right**).

**Figure 10:**
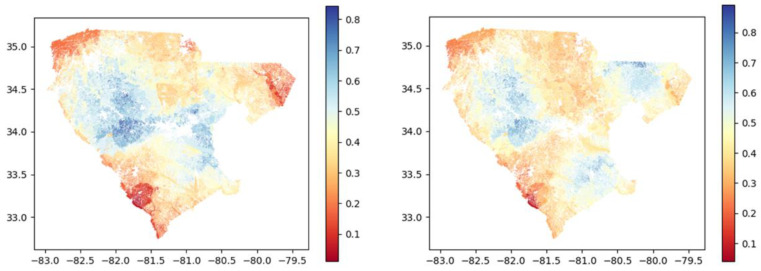
SSI integrated with transportation analysis using K-means (**left**) and graph theory (**right**).

Figure 9 shows the distribution of normalized scores for distances covered from fields to depots. In both graphs, distribution peaks appear to be the same, with most field scores within the 0.5–0.6 range. However, the distributions show differences. The K-means method (left) shows more scores closer to 0 (median value is 0.52 vs. 0.54 for the graph theory method) and fewer scores close to 1. This indicates that fields are generally farther from depots than the ones in the graph theory method (mean values of 0.52 vs. 0.54). Distances traveled are higher, illustrated by the right tail showing higher scores for the graph theory method (right). Figure 10 shows the region maps with SSI integrated using cluster analysis (left) and graph theory (right), respectively. Differences in the scores can be observed. The graph theory method seems to show more suitability around the depot locations. Because it presents more depots, transportation suitability scores are higher (as seen in Figure 9). Generally, the southwestern and northwestern parts are the least suitable, in both methods, illustrated by lower scores. The central parts show good suitability.

These differences might be explained by the number of depots and the difference in locations. Road networks are certainly different from one location to another. The changing distances from different points lead to different scores. The approaches in this study thus illustrate different supply chain configurations. Depending on where depots are located, and what the road network looks like in the area, a given configuration may be more cost-effective than the other. The graph theory approach presents a cumulative distance (from fields to depots in the respective service area) of 801,031,476 miles, while the K-means presents 1,037,606,072 miles, which is about 30% more miles traveled than in the graph method. This finding suggests that a more decentralized system may be more cost-effective, with depots located closer to more fields, if tying distance driven to transportation costs. This is if only transportation costs are considered, as it would be reasonable to assume that depot construction and running costs might be higher for three depots than just two. Those costs were not considered here. Also not considered were collection and delivery costs from depots to a biorefinery. There are also implications on gas emissions. The more driving there is, the more CO_2_ emission there is. A more centrally oriented supply chain design may thus provide a less sustainable option.

Biomass supply systems were modeled as a way to understand how feedstock can be produced and cost-effectively moved in the network for processing. Decentralized networks, which are geographically more spread out, tend to provide better reliability, as they offer more processing locations. With depots spread across multiple physical locations, the supply chain gains in resilience and resource sharing opportunities. From a redundancy perspective, more centralized architectures can be deficient in the event of a disruption. As these are highly dependent on network connectivity, the supply system becomes rapidly vulnerable if depots lose connectivity. A sustainable supply chain would require a more resilient architecture, with more depots and a reduced miles driven volume. The way forward lies in a more decentralized supply chain design and a more progressive holistic approach tapping into land suitability for natural resources to create sustainable systems. In using a clustering algorithm and coefficient, this study captures the topology of road networks and helps highlight the role of their various topological features in depot siting. By using two different approaches, we provide results that are more robust and compelling than with one approach. This is the main benefit of using multiple methods, as it offers a broader outlook on the issue at hand. Both methods are pertinent in the study of facility location problems (FLP), concerned with the placement of facilities to minimize transportation costs. However, it is important to note their differences and how these impact the analysis.

While the clustering coefficient (graph theory) relies on the actual road network connectedness and degree of accessibility, K-means uses the field centroid, independent of the road network, to form clusters [86]. The K-means method is dependent on initial values, which are a random choice of cluster centers. The rescaling of datasets may potentially change results and provide different conclusions. The selection criteria used in graph theory were also a defining factor in obtaining these results, as it uses the highest SSI value for fields to prioritize depots. Since SSI calculation uses a weighting approach with equal importance for all criteria, a different weighting approach could potentially provide a different ranking and ultimately different depot locations. In that sense, the results obtained are not to be interpreted as one method being necessarily better than the other, rather, they should provide insights on biomass supply chain designs.

By prioritizing *suitability* over *optimality* for depot selection, this study integrates ecological indicators and transportation network analysis and presents a scoring mechanism to assess the appropriateness of processing locations. Ecological indicators are measures of key ecosystem properties, providing information on pressures on the environment, environmental conditions, and societal responses [87]. As such, these are critical for implementing a suitability analysis. Performing a transportation network analysis provides information on network features and helps define movements and flows of feedstock. Integrating these analyses offers a more complete suitability assessment for biomass production and sustainability implications. The use of scoring for both analyses to synthesize underlying complexity help in generating and effectively communicating information about the biomass supply chain. In contrast to the more familiar optimization approach, the suitability approach is not intended to find the best or most optimal depot location, but to identify potential locations with respect to preprocessing. This involves the quantification of the subjective importance placed on various factors, opening a wide range of possibilities necessary for long-term planning. 

The transportation of products to pre-processing facilities or depots in supply chain analysis is recognized as one of the key components [88]. Facility location optimization substantially reduces transportation costs in the supply chain [89]. Different siting contributes to create different designs/architectures, with efficiency implications. As our results show, different designs resulting from depots differently distributed in the area of interest present different mileages driven, each of which incurs different costs and gas emissions. In this study, we show how ecological indicators of agricultural fields can and need to be integrated into the supply chain analysis. With the ILM approach, SRWC may be planted in environmentally adequate portions to protect soil resources by producing environmental and economic benefits, thereby improving the biomass supply–demand dynamics and making more feedstock available. This approach results in a landscape mosaic growing both conventional agricultural residues and dedicated energy crops at the same time, helping to implement a more sustainable biomass production scheme. Soil features representations afford, thus, a more realistic representation of the biomass supply chain, incorporating the conditions enabling cultivation and exploring the role of environmental factors via ecological indicators. This work makes progress toward a more advanced supply chain analysis, demonstrating that the sustainability of the biomass supply chain can be evaluated using suitability, measured by its capability to (1) develop a durable feedstock availability scheme and (2) offer a reliable transportation network enabling the conversion of raw biomass into a larger scale commodity feedstock.

This study can certainly be extended. Several factors including depot sizing and capacity, as well as feedstock blend components, can affect system costs [90]. Depot capacity, for instance, may be factored in to not overburden depot candidates and ultimately create an unfairly distributed network. Biomass quality is another factor having implications for the planning and design of the supply chain. Biomass quality, such as ash and moisture, impacts the overall cost and topology of the supply chain [91]. It would also be interesting to consider climate and its effects, as climate change is expected to impact soil properties and ecosystems [92]. The main limitation of this approach is in regard to the weighting, which we assume is equal across factors. This assumption does not account for the different needs of other crops on polyculture landscapes and also farmer preferences. A different weighting would provide different suitability scores.

## 4. Conclusions

The sustainability of the bioenergy supply in the US rests on the development of advanced approaches meant to inform renewable energy policy. Using geospatial data and heuristics, we investigated biomass supply chain design, leveraging soil characteristics relevant to SRWC and road networks. An integrated framework that models biomass production suitability and transportation throughout a biomass supply chain is presented. Biomass production was estimated based on ecological indicators facilitating growth, including land cover/crop rotation, slope, soil productivity, soil texture, and water availability. With successful ILM, SRWC may be planted in environmentally adequate portions to protect soil resources and improve the biomass supply–demand dynamics, making more feedstock available in the future.

This work proposes a different and innovative view of the biomass supply chain, proposing *suitability* rather than *optimality*. SSI scores were determined by conducting a site suitability analysis consisting of identifying, comparing, and ranking candidate fields more appropriate for SRWC. Transportation scores were obtained based on the location of depots, using two methods: graph theory and a clustering algorithm. Graph theory, via the clustering coefficient, helps to determine dense areas in the network and pinpoint the most appropriate depot location. The clustering algorithm, via K-means, helps discover grouping in clusters, classifying each field centroid into a specific cluster. The center of each cluster represents the location of the depot. A final score is then computed, using the average of SSI scores and transportation scores, estimating the overall supply chain suitability for SRWC.

While the K-means method found two depots, the graph theory method found three depots, resulting in a total of 1,037,606,072 and 801,031,476 miles driven from fields to depots, respectively. The distance covered by the former is about 1.3 times the distance covered by the latter, suggesting more decentralized systems to be more favorable to a sustainable supply chain, with the depots located closer to the fields. More decentralized-depot-based supply chain designs show more economical and environmental benefits in our region of interest. With depots spread across multiple physical locations, the supply chain system tends to be more robust in the event of disruptions.

The way forward for biomass supply chain analysis consists of a more holistic approach tapping into land suitability for natural resources to create sustainable systems. This work makes progress toward a more advanced supply chain analysis, demonstrating the use of suitability for biomass supply chain sustainability analysis. The approach presented lays out the foundations for discussion regarding scale-up potential and long-term viability of advanced bioenergy systems.

## Figures and Tables

**Table 2:** Factors considered for updated SSI.

Factors	Data/Assumptions
SSI: Index score quantifying the suitability of SRWC on fields located in the area of interest (defined in Section 2.3).	Computed using Equation (1).
Distance scoring: Index score quantifying distances from fields to depots.	Computed using a fuzzy rule. Distance values are indicated by a number in the range from 0 to 1, 1 scoring the fields that are closest to the depots, and 0 the farthest.

## Data Availability

All data used are available on links cited in the paper.
